# Changes in cellularity of Wistar rats bone marrow treated with fructose

**DOI:** 10.1186/1758-5996-7-S1-A130

**Published:** 2015-11-11

**Authors:** Tatiele Estefâni Schönholzer, Carolina Abreu Miranda, Betina Beatriz Mielke, Lusnaiara Rodrigues Lima, Kleber Eduardo de Campos, Paula Cristina de Souza Souto

**Affiliations:** 1 UFMT, Barra do Garças, Brazil

## Background

Fructose is a monosaccharide and is an important component of our diet. Children and young people consume foods low in nutrients and high in sugar, around 30% daily, drawing attention to the increase in obesity and chronic diseases in populations. During ontogeny, bone marrow supports the formation and development of cells. The functioning of the immune system depends on several nutrients that have important roles in the body, and its deficiency leads to depression of the immune system. It is relevant to study a young animal model before an intake of fructose, standardizing the porcentage used and the treatment time.

## Objective

The objective of this study was to evaluate the relationship of fructose diet and possible changes in cellularity of bone marrow of Wistar rats.

## Materials and methods

Wistar male rats were used. At 42 days of age they were divided in two groups: a) control group (C, n=7): rats that received standard diet and filtered water without addition of other substances b) Group fructose (F, n=8): rats which received standard diet and filtered water with addition of 7% fructose. The animals were weighed weekly. Was analyzed biochemical parameters for monitoring monthly blood glucose. At the end of treatment (day 90) they were sedated and euthanized. Was analyzed the immunological parameters of the bone marrow (bone marrow aspirate), for total cell count (Neubauer chamber) and differential count and myeloid/erythroid lineage. Statistical analysis was done through the Student t test, analysis of variance (ANOVA) and Multiple Comparison Test Dunnett. Values were considered statistically different when p ≤ 0.05.

## Results

The animals treated with fructose showed a hypercellular bone marrow with an increase in the erythroid and myeloid lineages (low neutrophils and leukocytosis) and also in myeloid/erythroid lineage. As for monthly blood glucose, the fructose group, there was a significant increase in the first month experiment with decrease in three months, compared to the control.

## Conslusion

The results showed that the percentage of fructose used was not sufficient to induce hyperglycemia in animals, but its ingestion resulted in low neutrophils and leukocytosis, howing that even at low doses fructose caused changes in hematopoiesis.

